# Signal Processing and Waveform Re-Tracking for SAR Altimeters on High Mobility Platforms with Vertical Movement and Antenna Mis-Pointing

**DOI:** 10.3390/s23229266

**Published:** 2023-11-18

**Authors:** Qiankai Wang, Wen Jing, Xiang Liu, Bo Huang, Ge Jiang

**Affiliations:** 1 Institute of Electronic Engineering, China Academy of Engineering Physics, Mianyang 621000, China; wangqiankai21@gscaep.ac.cn (Q.W.); x-liu12@foxmail.com (X.L.); vick123y@163.com (B.H.); jiangge321@163.com (G.J.); 2 Graduate School of China Academy of Engineering Physics, Mianyang 621000, China

**Keywords:** synthetic aperture radar altimetry, waveform re-tracking, least squares estimation

## Abstract

Synthetic aperture radar (SAR) altimeters can achieve higher spatial resolution and signal-to-noise ratio (SNR) than conventional altimeters by Doppler beam sharpening or focused SAR imaging methods. To improve the estimation accuracy of waveform re-tracking, several average echo power models for SAR altimetry have been proposed in previous works. However, these models were mainly proposed for satellite altimeters and are not applicable to high-mobility platforms such as aircraft, unmanned aerial vehicles (UAVs), and missiles, which may have a large antenna mis-pointing angle and significant vertical movement. In this paper, we propose a novel semi-analytical waveform model and signal processing method for SAR altimeters with vertical movement and large antenna mis-pointing angles. A new semi-analytical expression that can be numerically computed for the flat pulse response (FSIR) is proposed. The 2D delay–Doppler map is then obtained by numerical computation of the convolution between the proposed analytical function, the probability density function, and the time/frequency point target response of the radar. A novel delay compensation method based on sinc interpolation for SAR altimeters with vertical movement is proposed to obtain the multilook echo, which can optimally handle non-integer delays and maintain signal frequency characteristics. In addition, a height estimation method based on least squares (LS) estimation is proposed. The LS estimator does not have an analytical solution, and requires iterative solving through gradient descent. We evaluate the performance of the proposed estimation strategy using simulated data for typical airborne scenarios. When the mis-pointing angles are within 10 degrees, the normalized quadratic error (NQE) of the proposed model is less than 10^−10^ and the RMSE of *τ* obtained by the re-tracking method fitted by the proposed model is less than 0.2 m, which indicates the high applicability of the model and accuracy of the re-tracking method.

## 1. Introduction

Altimeters play a vital role in the effective operation of onboard platforms. The altitude data they provide supports critical functions such as maintaining stable flight levels, and plays an integral role in implementing advanced automated features. This includes the execution of autonomous return to launch points and automatic takeoff and landing processes. For instance, state-of-the-art consumer unmanned aerial vehicles (UAVs) utilize the Internal Measurement Unit (IMU) and Global Positioning System (GPS) to measure height. However, both sensors can only estimate the flight altitude relative to their starting position, and not the actual altitude over current terrain or vegetation [1]. Using radar sensors is advantageous, as they can directly sense range and velocity and are not affected by lighting conditions or contrast [2]. By integrating high-accuracy radar altimeters, the altitude measurement capabilities of onboard platforms can be significantly enhanced, enabling them to adapt more proficiently to dynamic flight conditions. This integration allows for the execution of intricate navigational tasks with heightened precision and dependability. Consequently, the importance of airborne altimeters is irrefutable in UAV operations [1,3] and aircraft takeoff and landing [4,5,6,7].

Radar altimeters can determine the distance between the radar and the ground surface. Pulse radar altimeters function by emitting a series of short pulses towards the ground and then measuring the time it takes for these pulses to be reflected back to the altimeter. The distance from the surface is determined by the round-trip delay of radar signals. The issue of high-accuracy altimetry has been extensively explored. The rough estimation of height can be obtained through tracking processing. Re-tracking is a crucial procedure applied in processing radar altimeter data to get more accurate height parameters [8]. It is an algorithm used to refine the estimated parameters of a radar echo, such as the time delay, power, and shape, which have been distorted by various factors during the signal’s round-trip travel from the altimeter to the ground surface and back. Re-tracking methods mitigate these distortions to obtain more accurate and reliable measurements. This is achieved by adjusting the parameters of the waveform model, which is representative of the idealized or expected shape of the radar echo. The re-tracking of altimeter echoes is commonly performed using statistical inference-based algorithms such as the Least Squares (LS) method [9,10,11,12], Maximum Likelihood Estimation (MLE) algorithm [13], and Bayesian inference [14,15]. The accuracy of altitude measurements from radar altimeters can be significantly improved through re-tracking.

To improve the estimation accuracy of waveform re-tracking, a series of average echo power models for SAR altimetry have been proposed. Brown made a series of assumptions about the scattering scenario of a flat sea surface in the 1970s, suggesting the seminal three-fold convolution form of the radar altimeter echo model. This model states that the echo power equals the convolution of the FSIR, point target response (PTR), and scatterer height density function (PDF) [16]. Based on the Brown model, a series of semi-analytical and analytical models have been developed to accommodate different scenarios [13,17,18]. SAR altimetry harnesses the Doppler properties of high pulse repetition frequency-transmitted pulses to enable a higher spatial resolution through along-track aperture synthesis [19]. SAR altimetry can mitigate speckle noise compared to conventional altimetry (CA) by permitting multiple observations of the same scatterers through synthetic aperture processing. The resulting shape of the multilook echo diverges from that of the CA echo, necessitating a new altimetric signal model. Numerous studies have introduced numerical models suited to SAR altimeters [20]. If SAR altimetry adheres to the same five assumptions as Brown’s model, the convolution model of traditional radar altimeters can be directly extended to it [21]. In analytical modeling of SAR altimeters, the sinc function obtained by coherent along-track accumulation is generally regarded as the sub-beam pattern after beam sharpening. Then, the sub-beam pattern is multiplied by the antenna pattern to establish the echo model according to the CA [16]. This extends the average flat pulse response in the average echo model to a two-dimensional description of the illumination response of different Doppler frequencies of sub-beams [21,22,23]. A rectangular function is used to approximate the sub-beam pattern; a semi-analytical model of the SAR altimeter echo was proposed based on the geometric approximation of the delay–Doppler bin in [22]. Their model was later enhanced by incorporating the effect of the antenna mis-pointing angle [12]. Furthermore, a Gaussian function was employed to approximate the sub-beam pattern, and the echo model included small antenna mis-pointing angles [23]. A closed-form expression for the SAR altimeter waveform was formulated in [24] by expressing the waveform in terms of a set of basis functions. An innovative processing scheme known as fully focused SAR (FF-SAR) was proposed in [25] to maximize the altimeter’s along-track resolution to the theoretical limit of half the antenna length, proving valuable in SAR imaging and radar altimetry. A new analytical derivation of a delay–Doppler map model for FF-SAR has been proposed, including vertical wave particle motion and a more general surface representation [26]. Unfocused SAR (UF-SAR) retracking methods (e.g., the physical SAMOSA model) have been utilized to retrack fully-focused SAR (FF-SAR) waveforms in [27], ultimately improving data quality for SAR altimetry in coastal regions. A novel coastal retracking algorithm for SAR altimetry was presented in [28] that employed an adaptive interference masking scheme to mitigate interfering signals from reflective coastal targets, significantly increasing the number of valid records in the coastal zone without compromising the quality of the estimated significant wave height.

Airborne altimeters frequently encounter skewed flight conditions and larger antenna mis-pointing angles, which is due to several reasons. Airborne platforms such as aircraft and drones are subject to constant changes in flight dynamics and conditions. Unlike satellites, which have a relatively stable trajectory in space, airborne vehicles often have to adjust their course and altitude to accommodate weather conditions, air traffic, and mission objectives. Such adjustments can lead to vertical movement. In addition, the maneuvers required for aircraft to follow specific survey paths or avoid obstacles can result in off-nadir pointing, leading to significant antenna mis-pointing angles. It must be emphasized that existing research has predominantly focused on altimeters in horizontal flight scenarios, and as such is not suitable for airborne scenarios. For instance, satellite altimeters such as CryoSat-2 generally consider mis-pointing angles of less than 1 degree barring specific maneuvering instances [29]. However, these angles can greatly exceed 1 degree during oblique flights due to substantial pitch and roll angles. Furthermore, mechanical vibrations and the inherent instability of aircraft flight can contribute to greater mis-pointing angles. Despite advancements in flight control systems, it remains challenging to maintain perfect alignment of the radar beam with the vertical axis [30]. All these factors are inherent to airborne operations, and present substantial challenges for high-accuracy altimetry based on echo data from airborne altimeters.

In this paper, we present a strategy for high-accuracy altimetry using airborne altimeters. We propose a generalized semi-analytical echo model for the re-tracking process of SAR altimetry with consideration of vertical movement and large antenna mis-pointing angles. This model encompasses a generalized analytical expression for the FSIR, which accounts for Doppler shift effects and distortions in illumination geometry induced by the flight path angle as well as for pronounced mis-pointing angles in oblique flight scenarios. The Gaussian approximation of the antenna mis-pointing angles is considered. The resulting delay–Doppler map (DDM) obtained by three-fold convolution is governed by six altimetric parameters: the epoch τ, significant wave height, Pu, flight path angle, and on-track and cross-track mis-pointing angles. Compared to satellite altimeters, the lower altitude of airborne altimeters results in a narrower Doppler beam, leading to a decrease in the precision of traditional delay compensation methods. A novel delay compensation method based on sinc interpolation for SAR altimeters with vertical movement is proposed to obtain the multilook echo, which can optimally handle non-integer delays and maintain the signal frequency characteristics. The validity of the proposed model is ascertained through the performance of the widely adopted least squares (LS) algorithm for altimetric parameter estimation [22,31]. We propose a five-parameter estimation strategy in which the antenna mis-pointing angle and flight path angle are updated with each iteration. The effectiveness of the proposed model and algorithm is demonstrated in ideal conditions, with the results confirming our model’s ability to accurately estimate the altimetric parameters.

## 2. Numerical Mean Echo Power Model for SAR Altimetry

**Figure 1:**
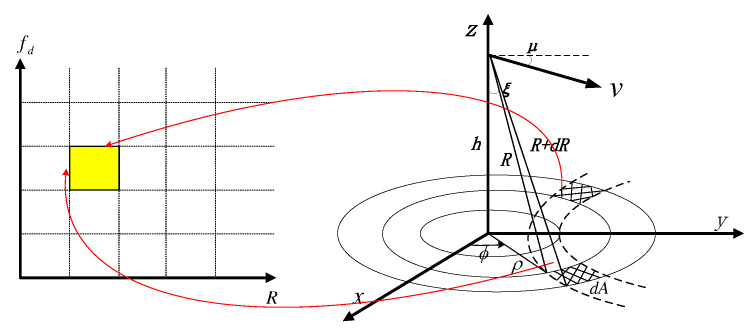
Delay–Doppler mapping. Each delay–Doppler bin is associated with two delay–Doppler cells on the surface.

Unlike CAs, which only perform power accumulation in range resolution bins, SAR altimeters transform the echo signal into the range–Doppler domain using the slow-time Fourier transform. The proposed model provides the semi-analytic expression of the mean signal power in each range–Doppler bin. As demonstrated in Figure 1, each range–Doppler bin is associated with two ground areas; the signal power can be expressed as the summation of the scatterers’ echo power from the two corresponding areas.

In this section, we derive a mean echo power model for an SAR altimeter with antenna mis-pointing and vertical movement. The altimetry scenario is introduced in Section 2.1. We first derive the echo signal from a single point target in Section 2.2, followed by pulse compression of the signal in Section 2.3 and the along-track FFT of the compressed signal in Section 2.4. Finally, the final numerical echo model of all the scatterers on the surface is provided in Section 2.5.

### 2.1. Altimetric Scenario

The altimetric scenario and coordinate system are shown in Figure 1, where the radar altimeter moves in the *y*–*z* plane at constant velocity *v* and incident angle μ. Here, the *x*–*y* plane corresponds to the flat surface illuminated by radar antenna, which can be considered flat for airborne applications, while the *z* axis is provided by the line from the nadir point on the ground to the radar antenna. Thus, the location of the radar at time *t* is provided by

(1) $$x_{r}\left(t\right)=0,\;y_{r}\left(t\right)=vt\cos\mu ,\;z_{r}\left(t\right)=h-vt\sin\mu ,$$

where *h* is the altitude when t=0.

Due to platform’s movement, the radar antenna may not look vertically downward towards the nadir point, and its beam directions are defined by angle ξ with respect to the *z* axis and angle $\tilde{\phi }$ with respect to the *x* axis. For simplification, we consider a Gaussian approximation of the radar antenna gain in the form

(2) $$G\left(\theta \right)=G_{0}e^{-\left(2/\gamma \right)\sin^{2}\theta },$$

where θ is the angle relative to the antenna’s boreside direction and γ determines the antenna’s beamwidth, which is provided by $\theta_{3\mathrm{dB}}=2\sin^{-1}\sqrt{(\gamma /2)\log2}$.

### 2.2. Radar Signal Model

In this section, we derive the echo signal from a single scatterer located at point (x,y,z) on the flat surface. To begin with, the radar transmit waveform is provided by

(3) $$s\left(t\right)=\sum_{n=0}^{N-1}s_{0}\left(t-nT_{r}\right)e^{j2\pi f_{0}t},$$

where $s_{0}\left(t\right)=1/\sqrt{T}rect\left(t/T\right)exp\left\{j2\pi f_{0}t+j\pi Kt^{2}\right\}$ is the baseband waveform in each pulse, $f_{0}$ denotes the carrier frequency, $T_{r}$ denotes the pulse repetition interval (PRI), and *N* is the number of pulses. Denoting the distance from the radar to the scatterer at time *t* by $R\left(t\right)$, the echo signal from this scatterer after down-conversion can be written as

(4) $$s_{r}\left(t\right)=\sum_{n=0}^{N-1}A_{0}s_{0}\left(t-nT_{r}-\frac{2R\left(t\right)}{c}\right)\exp\left(-j4\pi f_{0}\frac{R\left(t\right)}{c}\right),$$

where *c* is the propagation velocity, $A_{0}$ is a complex amplitude factor, and

$$R\left(t\right)=\sqrt{x^{2}+{\left(y-vt\cos\mu \right)}^{2}+{\left(h-z-vt\sin\mu \right)}^{2}}.$$

Let $t=t_{r}+nT_{r}$, where *n* is pulse index and $t_{r}$ denotes the radar’s fast time. Thus, the received signal during the *n*-th PRI is

(5) $$S_{r}\left(t_{r},n\right)=s_{r}\left(t_{r}+nT_{r}\right)=A_{0}s_{0}\left(t_{r}-\frac{2R\left(t_{r}+nT_{r}\right)}{c}\right)\exp\left(-j4\pi f_{0}\frac{R\left(t_{r}+nT_{r}\right)}{c}\right)$$

for n=0,…,N−1 and $0\leq t_{r}\leq T_{r}$.

### 2.3. Pulse Compression

We obtain the range profile of the scatterers via pulse compression. The signal after pulse compression in the *n*-th PRI is provided by

(6) $$\begin{array}{cc} C\left(t_{r},n\right) & =\int_{-\infty }^{\infty }S_{r}\left(\tau ,n\right)s_{0}^{*}\left(\tau -t_{r}\right)d\tau \\ \; & \approx A_{0}W_{r}\left(t_{r}-\frac{2R\left(t_{r}+nT_{r}\right)}{c}\right)\exp\left(-j4\pi f_{0}\frac{R\left(nT_{r}\right)}{c}\right), \end{array}$$

where $W_{r}\left(\cdot \right)$ is the autocorrelation function (ACF) of $s_{0}\left(t\right)$.

Before proceeding any further, applying approximations to the distance R(t) can be beneficial for simplifying the expression of $C\left(t_{r},n\right)$. For a small *N* and narrow antenna beam, we have

(7) $$R\left(t\right)\approx R_{0}-z-\frac{v\left(y\cos\mu +h\sin\mu \right)}{R_{0}}t=R_{0}-z-v_{r}t,$$

where $R_{0}=\sqrt{x^{2}+y^{2}+h^{2}}$ and $v_{r}=v\left(y\cos\mu +h\sin\mu \right)/R_{0}$ denotes the relative velocity of the scatterer with respect to the radar. Substituting (7) into the phase history in $C\left(t_{r},n\right)$ and assuming that the variation of R(t) during a coherent processing interval exceeds the range resolution of radar, we have

(8) $$C\left(t_{r},n\right)\approx A_{1}W_{r}\left(t_{r}-\frac{2\left(R_{0}-z\right)}{c}\right)\exp\left(j4\pi f_{0}\left(v_{r}/c\right)nT_{r}\right).$$

Here, the phase terms that are independent to *n* and $t_{r}$ are merged into the new factor $A_{1}=A_{0}\exp\left(j4\pi f_{0}\left(-R_{0}+z\right)/c\right)$. From (8), the Doppler frequency is provided by

(9) $$f_{d}=2v_{r}f_{0}/c=\frac{2vf_{0}\left(y\cos\mu +h\sin\mu \right)}{R_{0}c}.$$

### 2.4. Along-Track FFT

SAR altimeters can achieve higher along-track resolution through Doppler beam sharpening, which is accomplished using the inter-pulse Fourier transform. For convenience, we assume that *N* is an even number. Following discrete Fourier transformation, the signal output is

(10) $$\begin{array}{cc} \gamma \left(t_{r},k\right) & =\frac{1}{N}\sum_{n=0}^{N-1}C\left(t_{r},n\right)\exp\left(-j\frac{2\pi }{N}nk\right)\\ \; & =A_{2}W_{r}\left(t_{r}-\frac{2\left(R_{0}-z\right)}{c}\right)W_{a}\left(k-Nf_{d}T_{r}\right) \end{array}$$

for k=−N/2,…,N/2−1. Here, $A_{2}=A_{1}\exp\left[j\left(N-1\right)\pi \left(f_{d}T_{r}-k/N\right)\right]$ and the point target response $W_{a}\left(\cdot \right)$ in the Doppler domain is provided by

(11) $$W_{a}\left(k^{\prime }\right)=\frac{1}{N}\frac{\sin(\pi k^{\prime })}{\sin(\pi k^{\prime }/N)}\approx \mathrm{sinc}\left(k^{\prime }\right).$$

### 2.5. Backscattered Waveform Power Model

Following [6,16,24], the average backscattered power from a flat surface can be obtained by an integral of the echo signal power over all the scatterers on the surface. For a rough surface with roughness characterized by a height probability density p(z), the average power in the delay–Doppler bin $\left(t_{r},k\right)$ is

(12) $$P_{I}\left(t_{r},k\right)={\;\int \int \int \;}_{R^{3}}p\left(z\right)\frac{\lambda^{2}G^{2}\left(\theta \right)\sigma_{0}}{{\left(4\pi \right)}^{3}R_{0}^{4}L_{p}}PTR\left(t_{r}-2\left(R_{0}-z\right)/c,k-Nf_{d}T_{r}\right)\;dxdydz$$

when z≪h for k=−N/2,…,N/2−1 and $0\leq t_{r}\leq T_{r}$. Here, λ denotes the wavelength, $\sigma_{o}$ is the backscattering cross-section per unit scattering area, and $L_{p}$ is the two-way propagation loss. The point target response is

$$PTR\left(t_{r},k\right)={\left|W_{r}\left(t_{r}\right)W_{a}\left(k\right)\right|}^{2}.$$

In (12), G(θ), $R_{0}$, and $f_{d}$ all depend on *x* and *y*, while $\sigma_{0}$ is assumed to be independent of *x* and *y* for simplify.

Generally, it is not possible to provide a closed form result for the integration in (12). Nevertheless, a semi-analytic expression can be provided via the convolution model proposed in [16], as shown in the next section.

## 3. Semi-Analytical Echo Model of Airborne SAR Altimeters

In this section, we provide a semi-analytical echo model of airborne SAR altimeters through an approximation that reformulates $P_{I}\left(t_{r},k\right)$ into the following convolution:

(13) $$P_{I}\left(t_{r},k\right)=\frac{c}{2}p^{\prime }\left(t_{r}\right)*PTR\left(t_{r},k\right)*FSIR\left(t_{r},k\right).$$

Here, $p^{\prime }\left(t_{r}\right)=p\left(-ct_{r}/2\right)$ and $FSIR(t_{r},k)$ is the impulse response from a flat surface, which is defined in (17). The derivation of the convolution model is first provided in Section 3.1. Then, an approximate semi-analytical expression of $FSIR(t_{r},k)$ is provided in Section 3.2. The echo power after three-term convolution requires delay compensation to obtain multilook waveforms; thus, a novel delay compensation method is provided in Section 3.3. The multilook waveform model used for airborne SAR altimeters with antenna mis-pointing and vertical movement is characterized by six altimetric parameters: the significant wave height (SWH), epoch (τ), amplitude ($P_{u}$), flight path angle (μ), and on-track and cross-track mis-pointing angles ($\psi_{ac}$ and $\psi_{al}$, respectively). Finally, the effects of the mis-pointing angles on the multilook echoes are analyzed in Section 3.4.

### 3.1. Convolution Model

To obtain the convolution model in (13), we can first consider a flat surface with a small scale of roughness, namely, z≈0, for all the scatterers. Then, the average power in the delay–Doppler bin $\left(t_{r},k\right)$ can be provided by

(14) $$P_{FS}\left(t_{r},k\right)={\;\int \int \;}_{R^{2}}\frac{\lambda^{2}G^{2}\left(\theta \right)\sigma_{0}}{{\left(4\pi \right)}^{3}R_{0}^{4}L_{p}}PTR\left(t_{r}-2R_{0}/c,k-Nf_{d}T_{r}\right)\;dxdy$$

for k=−N/2,…,N/2−1 and $0\leq t_{r}\leq T_{r}$. We define $p^{\prime }\left(t_{r}\right)=p\left(-ct_{r}/2\right)$. It was proposed in [16] that $P_{I}\left(t_{r},k\right)$ can be expressed as a convolution between $p^{\prime }\left(t_{r}\right)$ and $P_{FS}\left(t_{r},k\right)$:

(15) $$P_{I}\left(t_{r},k\right)=\frac{c}{2}\int_{R}P_{FS}\left(t_{r}-\tau ,k\right)p^{\prime }\left(\tau \right)\;d\tau .$$

As it is difficult to determine the analytical expression of $P_{FS}\left(t_{r},k\right)$, we can consider an ideal point target response that is a delta function in the time domain and rectangular in the Doppler domain, namely, $PTR^{\prime }\left(t_{r},k\right)=\delta \left(t\right)I\left(k\right)$ and

(16) $$I\left(k\right)=\left\{\begin{array}{cc} 1, & |k|\leq 1/2,\\ 0, & |k|>1/2. \end{array}\right.$$

For $PTR^{\prime }\left(t_{r},k\right)$, $P_{FS}\left(t_{r},k\right)$ reduces to the impulse response from the flat surface

(17) $$FSIR\left(t_{r},k\right)={\;\int \int \;}_{R^{2}}\frac{\lambda^{2}G^{2}\left(\theta \right)\sigma_{0}}{{\left(4\pi \right)}^{3}R_{0}^{4}L_{p}}\delta \left(t_{r}-2R_{0}/c\right)I\left(k-Nf_{d}T_{r}\right)\;dxdy$$

for k=−N/2,…,N/2−1 and $0\leq t_{r}\leq T_{r}$.

The convolution between $FSIR\left(t_{r},k\right)$ and the actual point target response is

(18) $$\begin{array}{cc} \; & {\tilde{P}}_{FS}\left(t_{r},k\right)=\sum_{k^{\prime }=-\infty }^{+\infty }\int_{R}FSIR\left(t_{r}-\tau ,k-k^{\prime })PTR(\tau ,k^{\prime }\right)\;d\tau \\ \; & =\sum_{k^{\prime }=-\infty }^{+\infty }{\;\int \int \;}_{R^{2}}\frac{\lambda^{2}G^{2}\left(\theta \right)\sigma_{0}}{{\left(4\pi \right)}^{3}R_{0}^{4}L_{p}}PTR\left(t_{r}-2R_{0}/c,k^{\prime }\right)I\left(k-k^{\prime }-Nf_{d}T_{r}\right)\;dxdy. \end{array}$$

Using staircase approximation on the point target function

$$PTR\left(t_{r}-2R_{0}/c,k\right)\approx \sum_{k^{\prime }=-\infty }^{+\infty }PTR\left(t_{r}-2R_{0}/c,k^{\prime }\right)I\left(k-k^{\prime }\right),$$

we have ${\tilde{P}}_{FS}\left(t_{r},k\right)\approx P_{FS}\left(t_{r},k\right)$. Combining (15) and (18), we can finally write $P_{I}\left(t_{r},k\right)$ as a convolution of the height probability density, point target response, and impulse response from the flat surface, as shown in (13).

**Figure 2:**
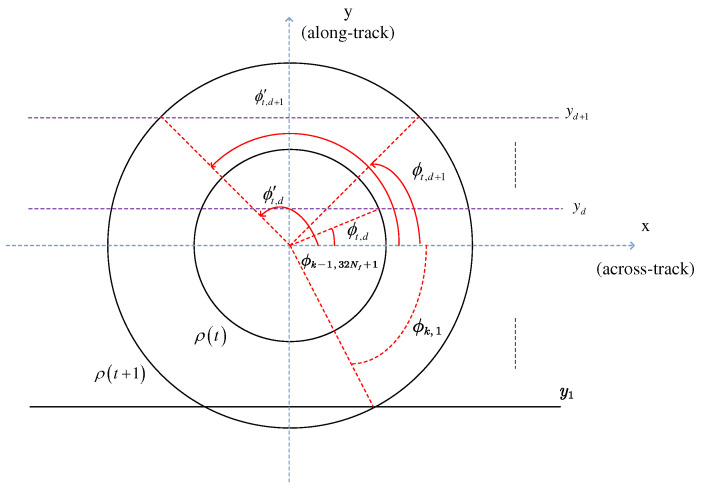
Circles of propagation and Doppler beams in SAR altimetry.

The corresponding FSIR is obtained by integrating over each Doppler beam. The *k*-th Doppler beam for time instant *t*, as shown in Figure 2, is defined by an integral angle ϕ varying within the range $D_{t_{r},k}=\left[\phi_{t_{r},k},\phi_{t_{r},k+1}\right]\cup \left[{\phi^{\prime }}_{t_{r},k},{\phi^{\prime }}_{t_{r},k+1}\right]$, leading to

(19) $$\phi_{t_{r},k}=\arcsin\left(y/\rho \left(t_{r}\right)\right)=\arcsin\left(\frac{f_{d}\lambda R_{0}+2v_{c}h\sin\mu }{2v_{c}\rho \left(t_{r}\right)\cos\mu }\right),$$

where $-2v\left(\rho \cos\mu +h\sin\mu \right)/\lambda R_{0}\leq f_{d}\leq -2v\left(h\sin\mu -\rho \cos\mu \right)/\lambda R_{0}$; here, the parameters xy in $f_{d}$ are omitted for brevity. Note that in the absence of antenna mis-pointing the two delay–Doppler bins exhibit symmetry along the track, resulting in equal power contributions to the DDM. However, their contributions differ when mis-pointing is considered, resulting in

(20) $$\phi_{t_{r},k}=\pi -{\phi^{\prime }}_{t_{r},k+1}\;\mathrm{and}\;\phi_{t_{r},k+1}=\pi -{\phi^{\prime }}_{t_{r},k}.$$

In SAR altimetry, the integral area of each Doppler beam varies due to the Doppler effect. The integration interval corresponding to the *k*-th Doppler beam is denoted as $D_{t_{r},k}$, resulting in

(21) $$FSIR\left(t_{r},k\right)=\frac{\lambda^{2}\sigma^{0}}{{\left(4\pi \right)}^{3}L_{p}}\int_{R^{+}\times D_{t_{r},k}}\frac{\delta \left(t_{r}-2R_{0}/c\right)G^{2}\left(\rho ,\phi \right)}{r^{4}}\rho d\rho d\phi .$$

Using $r=h\sqrt{1+\varepsilon^{2}}$, we obtain

(22) $$FSIR\left(t_{r},k\right)=\frac{\lambda^{2}\sigma^{0}G_{0}^{2}}{{\left(4\pi \right)}^{3}L_{p}h^{4}}\int_{R^{+}\times D_{t_{r},k}}\frac{\delta \left(t_{r}-2R_{0}/c\right)\exp\left\{-(\frac{4}{\gamma }\sin^{2}\left[\theta \left(\rho ,\phi \right)\right])\right\}}{{\left(1+\varepsilon^{2}\right)}^{2}}\rho d\rho d\phi$$

with

(23) $$\cos\left[\theta \left(\rho ,\phi \right)\right]=\frac{\cos\left(\xi \right)+\frac{\rho }{h}\sin\left(\xi \right)\cos\left(\tilde{\phi }-\phi \right)}{\sqrt{1+\frac{\rho^{2}}{h^{2}}}}.$$

### 3.2. Semi-Analytical Expression of $FSIR(t_{r},k)$

Using the change of variable $\begin{array}{c} x=\left(2h/c\right)\sqrt{1+\varepsilon^{2}} \end{array}$ and the property of the Dirac distribution $h\left(t\right)=\int_{c_{1}}^{+\infty }\delta \left(x-t\right)h\left(x\right)dx$ for $t\geq c_{1}$, integrating with respect to *x* leads to

(24) $$\begin{array}{c} FSIR\left(t_{r},k\right)=\frac{P_{u}}{2\pi }{\left(1+\frac{ct}{2h}\right)}^{-3}J\left(t\right)\times \left\{\int_{D_{t_{r},k}}F\left[\tilde{\phi }-\phi ,\varepsilon ,\xi ,\mu \right]\;d\phi \right\} \end{array}$$

with

$$\begin{array}{c} F\left[\tilde{\phi }-\phi ,\varepsilon ,\xi ,\mu \right]=\exp\left\{-4\left[1-\cos^{2}\left(\xi \right)/\left(1+\varepsilon^{2}\right)\right]/\gamma \right.\left.+b\left(1-\sin^{2}\left(\tilde{\phi }-\phi \right)\right)+a\cos\left(\tilde{\phi }-\phi \right)\right\} \end{array},$$

ε=ρ/h,

$$P_{u}=\frac{\lambda^{2}G_{0}^{2}c\sigma^{0}}{4{\left(4\pi \right)}^{2}L_{p}h^{3}},$$

$$a=\frac{4\varepsilon \sin\left(2\xi \right)}{\gamma \left(1+\varepsilon^{2}\right)},$$

(25) $$b=\frac{4\varepsilon^{2}\sin^{2}\left(\xi \right)}{\gamma \left(1+\varepsilon^{2}\right)},$$

where $J\left(\cdot \right)$ is the step function and $D_{t,k}$ and $\varphi_{t,k}$ are both provided in (19). Performing a variable conversion $w=\tilde{\phi }-\phi$ in (24) results in

(26) $$\begin{array}{c} \mathrm{FSIR}\left(t_{r},k\right)=\frac{P_{u}}{2\pi }{\left(1+\frac{ct}{2h}\right)}^{-3}J\left(t\right)\times \exp\left\{-4\left[1-\cos^{2}\left(\xi \right)/\left(1+\varepsilon^{2}\right)\right]/\gamma +b/2\right\}\\ \times \left[f\left(\tilde{\phi }-\phi_{t_{r},k+1},\tilde{\phi }-\phi_{t_{r},k}\right)+f\left(\tilde{\phi }-{\phi^{\prime }}_{t_{r},k+1},\tilde{\phi }-{\phi^{\prime }}_{t_{r},k}\right)\right] \end{array}$$

with

(27) $$f\left(w_{1},w_{2}\right)=\int_{u_{1}}^{u_{2}}\exp\left[a\cos\left(w\right)+\frac{b}{2}\cos\left(2w\right)\right]\;dw,$$

where $f\left(\cdot \right)$ can be expanded using Bessel functions as follows:

$$\exp\left[a\cos\left(w\right)\right]=I_{0}\left(a\right)+2\sum_{k=1}^{\infty }I_{k}\left(a\right)\cos\left(kw\right)$$

(28) $$\exp\left[\frac{b}{2}\cos\left(2w\right)\right]=I_{0}\left(\frac{b}{2}\right)+2\sum_{k=1}^{\infty }I_{k}\left(\frac{b}{2}\right)\cos\left(2kw\right),$$

where $I_{k}$ denotes the first-kind modified Bessel function of order *k*. Note that if any of these terms are approximated, the higher-order expansions of $\exp\left[\frac{b}{2}\cos\left(2w\right)\right]$ can lead to an unacceptable increase in model error when the mis-pointing angle exceeds 1 degree. In this paper, the complete analytical form of the Bessel function approximation is obtained without neglecting any terms. Subsequently, upon substituting (28) into (27) and simplifying, we have

(29) $$\begin{array}{cc} f\left(w_{1},w_{2}\right) & =\sum_{k=0}^{\infty }\sum_{j=0}^{\infty }C_{k}C_{j}I_{k}\left(a\right)I_{j}\left(\frac{b}{2}\right)/2\times \{w_{2}\left[\mathrm{sinc}\left(\left(k-2j\right)w_{2}\right)-\mathrm{sinc}\left(\left(k+2j\right)w_{2}\right)\right]\\ \; & -w_{1}\left[\mathrm{sinc}\left(\left(k-2j\right)w_{1}\right)-\mathrm{sinc}\left(\left(k+2j\right)w_{1}\right)\right]\} \end{array},$$

where $C_{k},C_{j}=\left\{\begin{array}{c} 1,k=0\\ 2,k\geq 1 \end{array}\right.$. The infinite sum provided in (29) can be truncated to reduce computational complexity by maintaining a finite number *m* of elements based on the accuracy requirement, as follows:

(30) $$\begin{array}{cc} f_{1}\left(w_{1},w_{2}\right) & =\sum_{k=0}^{m}\sum_{j=0}^{m}C_{k}C_{j}I_{k}\left(a\right)I_{j}\left(\frac{b}{2}\right)/2\times \{w_{2}\left[\mathrm{sinc}\left(\left(k-2j\right)w_{2}\right)-\mathrm{sinc}\left(\left(k+2j\right)w_{2}\right)\right]\\ \; & -w_{1}\left[\mathrm{sinc}\left(\left(k-2j\right)w_{1}\right)-\mathrm{sinc}\left(\left(k+2j\right)w_{1}\right)\right]\}. \end{array}$$

Finally, the analytical expression of FSIR is provided by

(31) $$\begin{array}{c} \mathrm{FSIR}\left(t_{r},k\right)=\frac{P_{u}}{2\pi }{\left(1+\frac{ct}{2h}\right)}^{-3}J\left(t\right)\times \exp\left\{-4\left[1-\cos^{2}\left(\xi \right)/\left(1+\varepsilon^{2}\right)\right]/\gamma +b/2\right\}\\ \times \left[f_{1}\left(\tilde{\phi }-\phi_{t_{r},k+1},\tilde{\phi }-\phi_{t_{r},k}\right)+f_{1}\left(\tilde{\phi }-{\phi^{\prime }}_{t_{r},k+1},\tilde{\phi }-{\phi^{\prime }}_{t_{r},k}\right)\right]. \end{array}$$

### 3.3. Multilooking

The reflection power is obtained through the triple convolution in (13), where the expressions of $FSIR(t_{r},k)$ are provided in (31). To obtain multilook waveforms, delay compensation can be applied to the DDM [19]. The range of each beam *n* needs to be offset for a particular range bin in order to correspond to the same across-track position for all beams [24]. After compensation, the range indicated for each scatterer over its entire illumination history is equal to its minimum range. The delay of each beam $\delta_{r}\left(n\right)$ is obtained by the difference between the modulus of the position vector $r_{n}=\sqrt{h^{2}+y_{n}^{2}}$ and the minimum height between the surface and the platform, as follows:

$$\delta_{r}\left(n\right)=r_{n}\left(1/\sin\theta_{n}-1\right),$$

(32) $$\cos\theta_{n}=\frac{\lambda f_{n}}{2v_{c}\cos\mu }+\frac{h_{0}\tan\mu }{r_{n}},$$

where the minimum height $h_{0}$ can be obtained by tracking algorithm. Then, the final multilook waveform can be obtained through the reflected power after delay compensation, as follows:

(33) $$s\left(t\right)=\sum_{n=1}^{N}P\left(t-2\delta_{r}\left(n\right)/c,f_{n}\right).$$

The traditional method for delay compensation in SAR altimetry, as described in [19], typically employs nearest neighbor interpolation in the range domain. In this paper, a novel delay compensation method for SAR altimeters with vertical movement is proposed. The proposed method utilizes sinc interpolation, as depicted in Algorithm 1, to optimally handle non-integer delays and maintain the frequency characteristics of the signal. The resulting multilook waveform is tracked according to the maximum value to find the range gate. We compare the tracked waveform to the proposed theoretical echo model in order to determine the parameter value exhibiting the highest similarity through the corresponding re-tracking algorithm detailed in Section 4.
**Algorithm 1** Delay Compensation for Airborne SAR Altimetry**Input:** range *r*, Doppler frequency $f_{d}$, The reflected power $P_{I}\left(r,f_{d}\right)$, minimum height $h_{0}$ 1:resolution of the along-track dy=vcosμNPRI; 2:$\mathrm{ylimit}=\lambda f_{d,\max}r_{\max}/2v\cos\mu$; 3:$y_{n}=-\mathrm{ylimit}+h\tan\mu :dy:\mathrm{ylimit}+h\tan\mu$; 4:**for** each *i* in *r* **do** 5:  $f_{n}$ according to (9); 6:  $P\left(r,f_{n}\right)=Interpolation\left(f_{d},P\left(r,f_{d}\right),f_{n}\right)$; 7:**end for** 8:$r_{n}=\sqrt{r\cdot r+{\left(y_{n}\cdot y_{n}\right)}^{T}}$ 9:**for** each *n* in $y_{n}$ **do**10:  $P\left(r_{n},f_{n}\right)=Interpolation\left(r,P\left(r,f_{n}\right),r_{n}\right)$;11:**end for****Output:** The reflected power after delay compensation $P\left(r_{n},f_{n}\right)$

### 3.4. Analysis of the Semianalytical Echo Model

**Figure 3:**
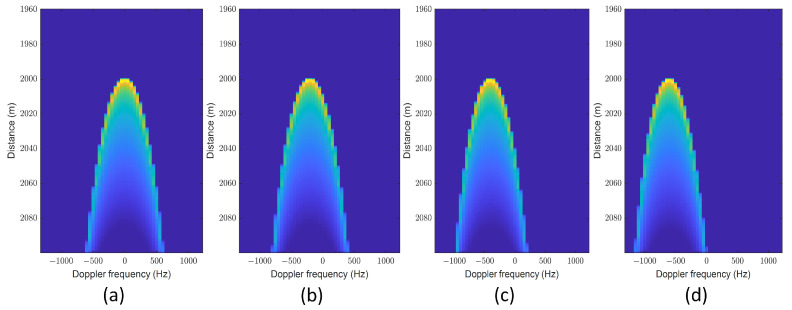
Effect of the flight path angle on the DDM: (**a**) $\mu =0^{o}$; (**b**) $\mu =6^{o}$; (**c**) $\mu =12^{o}$; (**d**) $\mu =18^{o}$ (Pu = 1, τ = 30 gates, SWH = 2 m, and $\psi_{ac}=0^{o}$).

**Figure 4:**
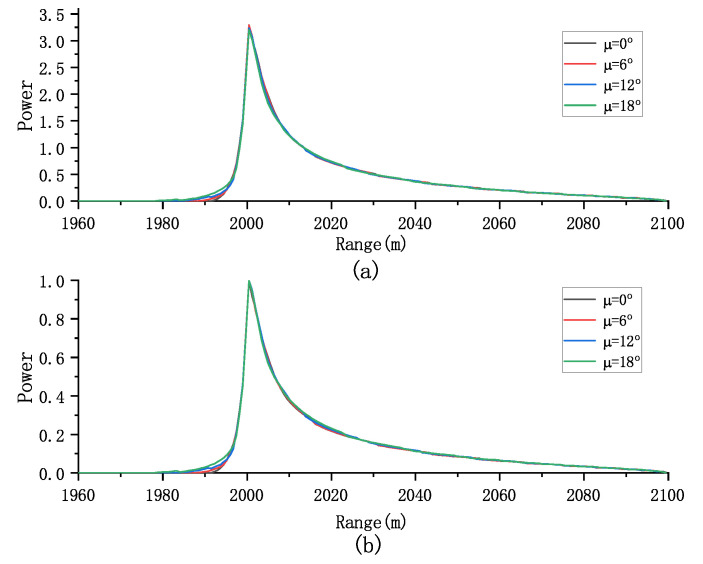
Effect of flight path angle on (**a**) the multilook echoes and (**b**) the normalized multilook echoes (Pu = 1, τ = 30 gates, SWH = 2 m, and $\psi_{al}=\psi_{ac}=0^{o}$).

The model aggregates the backscattered energies within the illuminated region to obtain a 2D FISR. The temporal dimension is determined by the propagating circle, while the frequency dimension is defined by the rectangular Doppler beam [22]. Each circle with a radius $\rho \left(t\right)=\sqrt{hct}$ and each approximate rectangular Doppler beam divides the illuminated area into distinct delay/Doppler bins. Note that the flight path angle μ introduces a Doppler frequency shift when the airborne platform flies obliquely (see Figure 3). As μ increases, all Doppler beams exhibit a negative Doppler shift $f_{d,shift}=-2v_{c}h\sin\mu /\lambda r_{xy}$. Additionally, the power within each delay/Doppler bin changes, deviating from a symmetric distribution. Due to disparities in offset range among different delay–Doppler bins after multilooking, the leading edge of the multilook waveforms is correspondingly affected, as shown in Figure 4.

However, changes in the velocity direction of the airborne platform generally result in antenna mis-pointing angles. Although the mapping relationship is unchanged when considering ξ and $\tilde{\phi }$, the backscattered energy within the delay–Doppler bins is impacted. In the case where the antenna is vertically fixed to the aircraft, the two mis-pointing angles ξ and $\tilde{\phi }$ are dependent on the pitch angle $\psi_{\mathrm{al}}$ and the roll angle $\psi_{\mathrm{ac}}$ as follows:

(34) $$\begin{array}{c} \psi_{\mathrm{al}}=\arctan\left(\tan\left(\xi \right)\sin\left(\tilde{\phi }\right)\right)\\ \psi_{\mathrm{ac}}=\arctan\left(\tan\left(\xi \right)\cos\left(\tilde{\phi }\right)\right). \end{array}$$

**Figure 5:**
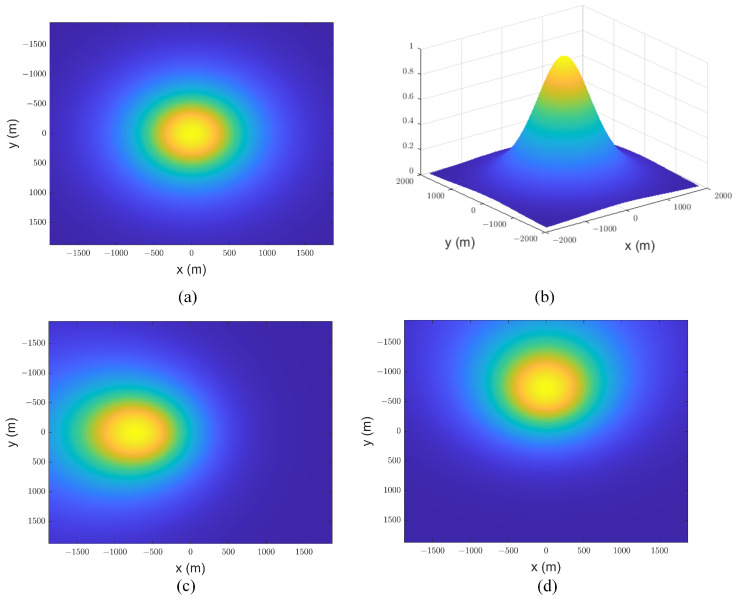
Antenna gain with different mis-pointing angles: (**a**) $\xi =0^{o},\hat{\phi }=0^{o}$; (**b**) $\xi =0^{o},\hat{\phi }=0^{o}$; (**c**) $\xi =20^{o},\hat{\phi }=0^{o}$; (**d**) $\xi =20^{o},\hat{\phi }=90^{o}$.

Figure 5 illustrates the variations in antenna gain across the various mis-pointing angles. It can be observed that the maximum antenna gain is achieved at the nadir point when the antenna mis-pointing angle is zero. As expected, the maximum antenna gain shifts along either the x or y axis as the antenna mis-pointing angle changes (see Figure 5c,d). The corresponding multilook echoes are significantly influenced by the variations in antenna gain. Next, we analyze the impact of antenna gain variation on multilook echoes.

**Figure 6:**
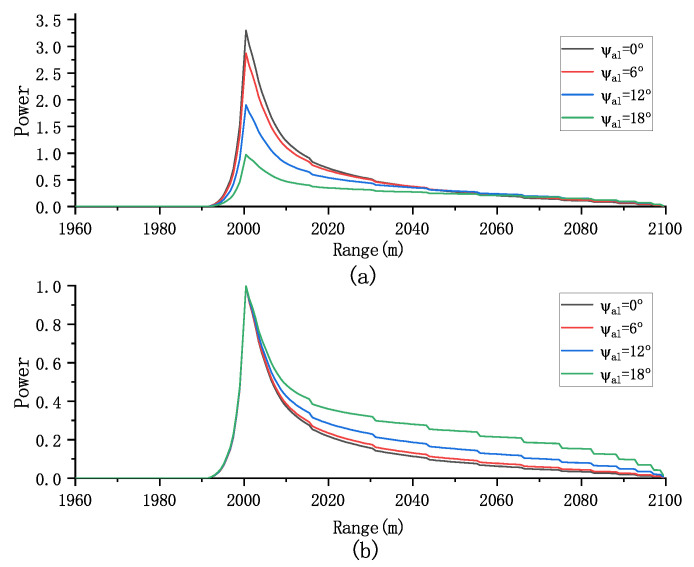
Effect of $\psi_{al}$ on (**a**) the multilook echoes and (**b**) the normalized multilook echoes (Pu = 1, τ = 30 gates, SWH = 2 m, and $\mu =\psi_{al}=0^{o}$).

We can evaluate the effect of antenna mis-pointing angle on both the multilook and normalized multilook echoes. As shown in Figure 6, increasing the mis-pointing angle along the track leads to a decrease in the amplitude of the multiview echo. However, this has a minimal effect on the normalized multiview echo, indicating that the mis-pointing angle along the track primarily affects the echo’s amplitude rather than its shape.

**Figure 7:**
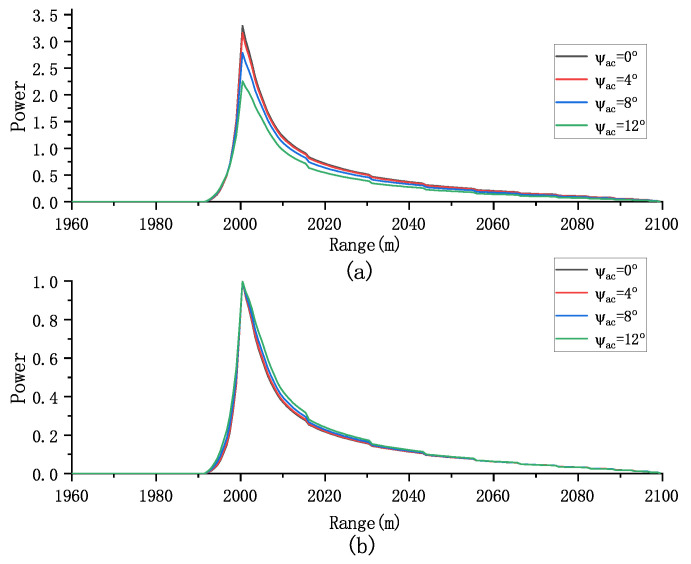
Effect of $\psi_{ac}$ on (**a**) the multilook echoes and (**b**) the normalized multilook echoes (Pu = 1, τ = 30 gates, SWH = 2 m, and $\mu =\psi_{ac}=0^{o}$).

In addition, Figure 7 demonstrates that the mis-pointing angle across the track has a more pronounced impact on the waveform’s amplitude compared to the along-track mis-pointing angle, particularly affecting the trailing edge of the waveform. Note that the mis-pointing echoes obtained by the proposed model are influenced by six parameters $\left(P_{u},\mathrm{SWH},\tau ,\mu ,\psi_{ac},\psi_{al}\right)$. The waveform’s shape is influenced by both the antenna mis-pointing and flight path angles, which should be considered in parameter estimation. Therefore, our focus is on analyzing the effect of the parameter vector $\left(\mathrm{SWH},\tau ,\mu ,\psi_{ac},\psi_{al}\right)$ on the normalized echo.

## 4. Re-Tracking Algorithm Based on Echo Model Fitting

Re-tracking processing involves parameterizing the echo function curve based on the rough tracking result. It uses a certain algorithm to fit the echo model to the actual waveform to achieve the most consistent state, thereby retrieving the parameters contained in the echo. It can be solved using the hill-climbing algorithm [32,33] or the least squares (LS) method [34]. However, in certain scenarios the paths defined by the hill-climbing algorithm do not monotonically reduce the distance to the local optimum [35]. Thus, the nonlinear least squares (LS) algorithm is widely used in altimetry parameter estimation. The LS estimator under consideration is defined as

(35) $${\hat{\theta }}_{LS}=\underset{\theta }{\arg\min}\frac{1}{2}\sum_{k=1}^{K}g_{k}^{2}\left(\theta \right),$$

where $g_{k}\left(\theta \right)=y_{k}-s_{k}\left(\theta \right)$ is the residual vector, $s\left(\theta \right)$ represents the multilook echoes, and $y={\left(y_{1},\ldots ,y_{K}\right)}^{T}$ is the echo destroyed by speckle noise. Because $g_{k}^{2}\left(\theta \right)$ is a complicated nonlinear function of SWH and τ, the optimization problem does not admit a closed-form expression, and the LS estimator (35) is solved using a numerical optimization method.

**Figure 8:**
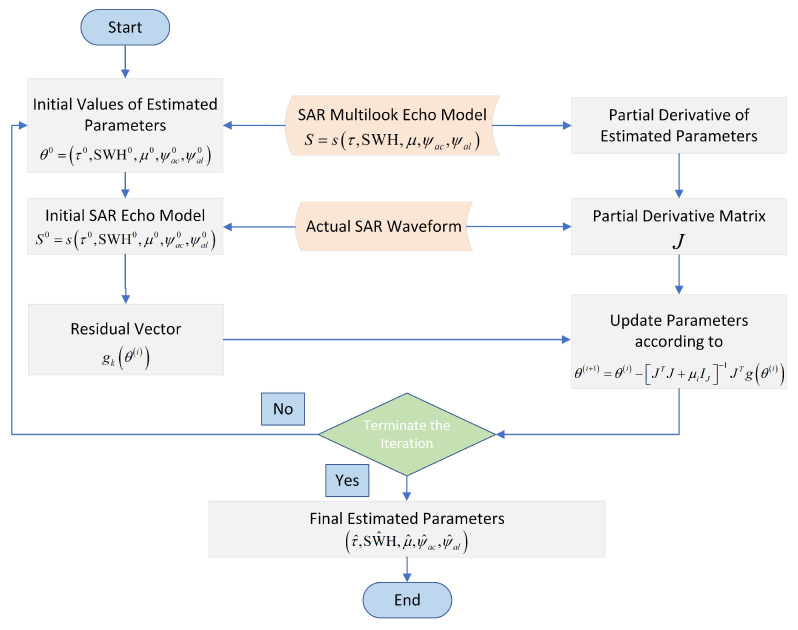
Flowchart of re-tracking algorithm implementation step.

The Levenberg–Marquardt (LM) algorithm is an optimization algorithm used for nonlinear least squares fitting and optimization [12,22,36]. It exhibits greater robustness and effectiveness than traditional LS methods. In this paper, a five-parameter iterative LS algorithm based on the LM algorithm is used to estimate the altimetric parameters, as shown in Figure 8.

The parameter update of the iterative LM algorithm is defined by $\theta^{\left(i+1\right)}=\theta^{\left(i\right)}+e^{\left(i\right)}$, where $\theta^{i}$ is the estimate of θ at the *i*-th iteration. The choice of $e^{i}$ is based on Taylor expansion (at the first order) of *g* in the neighborhood of $\theta^{i}$

(36) $$g\left(\theta^{\left(i\right)}+e^{\left(i\right)}\right)=g\left(\theta^{\left(i\right)}\right)+J\left(\theta^{\left(i\right)}\right)e^{\left(i\right)}$$

where $J=\left[\partial g\left(\theta^{\left(i\right)}\right)/\partial \theta_{1},\ldots ,\partial g\left(\theta^{\left(i\right)}\right)/\partial \theta_{J}\right]$ is a partial differential matrix representing the gradient of *g*. After replacing (36) in (35), the following result is obtained:

(37) $$G\left(\theta +e\right)≃L\left(e\right)=\frac{1}{2}l{\left(e\right)}^{T}l\left(e\right).$$

The descent direction *e* is then obtained by minimizing $L\left(e\right)$. By setting the derivative $L^{\prime }\left(e\right)=J{\left(\theta \right)}^{T}g+J{\left(\theta \right)}^{T}J\left(\theta \right)e$ to zero and adding a regularization parameter μ, leading to

(38) $$\left[J{\left(\theta \right)}^{T}J\left(\theta \right)e+\mu I_{J}\right]e=-J{\left(\theta \right)}^{T}g$$

where $I_{J}$ is the identity matrix, this algorithm employs a gradient descent method to update the parameters $\theta =\left(\mathrm{SWH},\tau ,\mu ,\psi_{ac},\psi_{al}\right)$, as follows:

(39) $$\theta^{\left(i+1\right)}=\theta^{\left(i\right)}-{\left[J^{T}J+\mu_{i}I_{J}\right]}^{-1}J^{T}g\left(\theta^{\left(i\right)}\right)$$

where $\mu_{i}$ controls the convergence speed of the algorithm. Note that the derivatives appearing in $\frac{\partial g\left(\theta \right)}{\partial \theta_{J}}$ can be computed numerically using the finite difference, as follows:

(40) $$\frac{\partial g\left(\theta \right)}{\partial \theta_{j}}≃-\frac{s\left(\theta_{j}+\Delta \theta_{j}\right)-s\left(\theta_{j}\right)}{\Delta \theta_{j}}.$$

It should be noted that the initial value of the iterated parameters in the re-tracking algorithm is $\theta^{0}=\left(\tau^{0},\mathrm{SW}H^{0},\mu^{0},\psi_{ac}^{0},\psi_{al}^{0}\right)$, where $\tau^{0}$ is obtained by rough tracking. Furthermore, the real mis-pointing angles (i.e., the roll and pitch angles) are used as inputs, deviating from their actual values by approximately $1^{\circ }$. These mis-pointing angles are recorded by the IMU during airborne experiments, and the deviation in the input mis-pointing angles depends on the precision of the IMU devices. This enhances the fitness between the theoretical echo model and the actual airborne waveform, thereby reducing the possibility of tracking failure caused by the low similarity between the theoretical model and actual waveform [12,37].

## 5. Results

In this section, we present the simulation results obtained with the proposed model. First, the approximations used to obtain the analytical FSIR are justified in Section 5.1. Then, the accuracy of the proposed model is evaluated through comparison with the fully numerical model in Section 5.2. Finally, in Section 5.3 we verify the effectiveness of the proposed estimation algorithm by comparing it with an algorithm that does not consider mis-pointing angles.

### 5.1. Analysis of the FSIR Approximations

The purpose of this section is to quantitatively analyze the error of the proposed model caused by the m-order approximation used in FSIR. The multilook echoes obtained from the proposed model are analyzed to examine the effect of different antenna mis-pointing angles and varying numbers of terms *m*. The validation involves comparing the model (13) with the numerical calculation (21). The approximation error of the proposed model is evaluated using the normalized quadratic error (NQE), which is defined as follows:

(41) $$\mathrm{NQE}\left(s,r\right)=\sqrt{\frac{\sum_{k=1}^{K}{\left(s_{k}-r_{k}\right)}^{2}}{\sum_{k=1}^{K}{r_{k}}^{2}}}.$$

The approximation of the FSIR is affected by the mis-pointing angle of the antenna and the number of terms *m*. Considering the use of normalized waveforms in the re-tracking algorithm, it is necessary to explain the relationship between the maximum error of the echoes and the number of terms *m*. NQE is used to describe the error of the maximum value of the echo $\mathrm{NQ}E_{\max}=\sqrt{{\left(s_{\max}-r_{\max}\right)}^{2}/r_{\max}^{2}}$.

**Figure 9:**
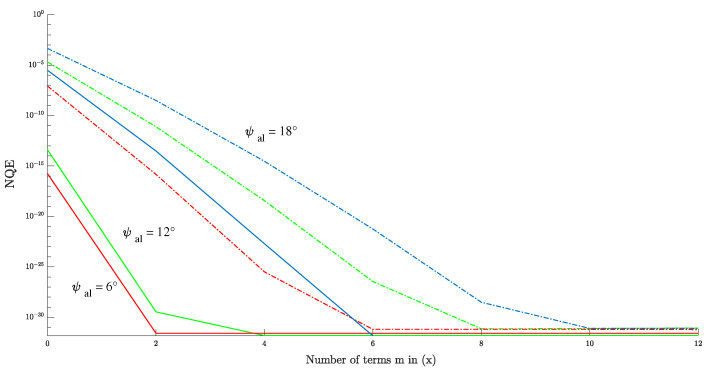
Overallerror versus *m* for different $\psi_{ac}$, showing the global NQE (continuous line) and NQE of echo maximum (crossed line) for $\psi_{ac}=6^{o}$ (in red), $\psi_{ac}=12^{o}$ (in green), and $\psi_{ac}=18^{o}$ (in blue).

Figure 9 shows the results of the NQE of the semi-analytical model as it changes with $\psi_{ac}$. It can be seen that NQE increases as the mis-pointing angle $\psi_{ac}$ becomes larger. When considering $\psi_{ac}\leq 18^{\circ }$ ($\psi_{ac}<\theta_{3\mathrm{dB}}/2$), m=4 is adequate to achieve the required level of error.

**Figure 10:**
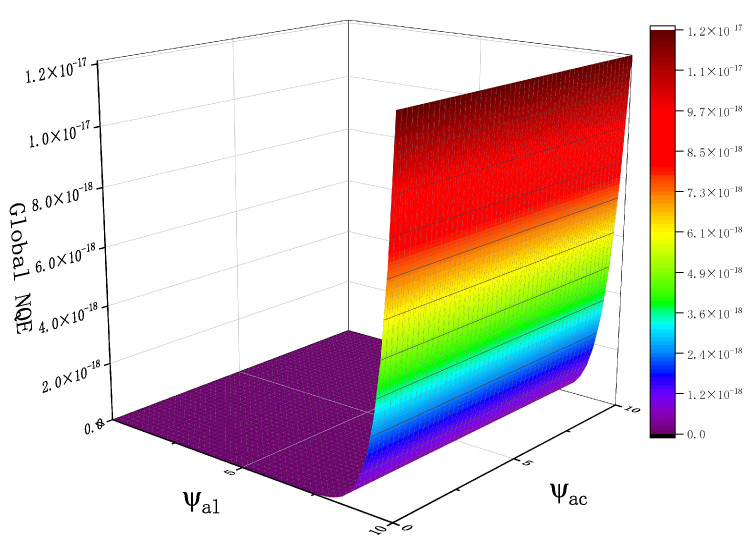
Overall error versus $\psi_{ac}$ and $\psi_{al}$ when m=4 (Pu = 1, τ = 30 gates, SWH = 2 m, and $\mu =0^{o}$).

For the case in which m=4, the global NQE matrix of the antenna mis-pointing angle is shown in Figure 10. Note that the NQE of a noisy echo may increase in real application, which requires *m* to take a larger value in order to reduce the error.

### 5.2. Comparison with Numerical Waveforms

The proposed semi-analytical model reduces the high dimensionality of the proposed numerical model by introducing approximations into the provided impulse response under the hypothesis of larger variation of the mis-pointing angles. We performed echo simulation in typical airborne scenarios; the simulation parameters are shown in Table 1. The multilook waveform obtained by the proposed model is considered to be a DDA echo, while the multilook waveform corresponding to the numerical integral form of (14) is considered to be a numerical echo. By comparing the DDA echo and numerical echo, we aim to assess how the accuracy of the semi-analytical model decreases as the mis-pointing angles increase. In addition, the semi-analytical model with all mis-pointing angles at zero is defined as G-DDA for comparison. Through comparison with the generalized model, we can evaluate the accuracy of the semianalytical waveforms with respect to the fully numerical waveforms for different values of the mis-pointing angles and flight path angle.

**Figure 11:**
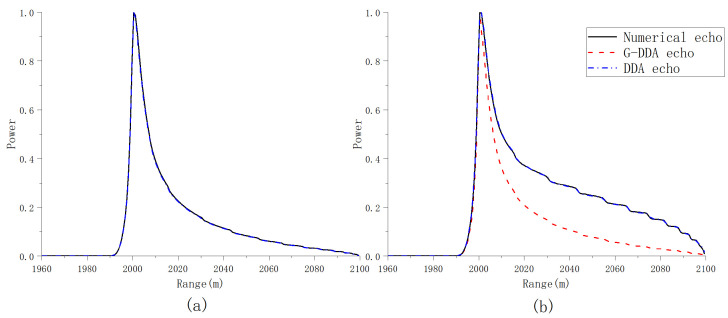
Comparison of multilooked power waveforms in typical airborne scenarios: (**a**) $\left(\mu ,\psi_{ac},\psi_{al}\right)=\left(0,0,0\right)\deg$ and (**b**) $\left(\mu ,\psi_{ac},\psi_{al}\right)=\left(18,10,18\right)\deg$.

**Figure 12:**
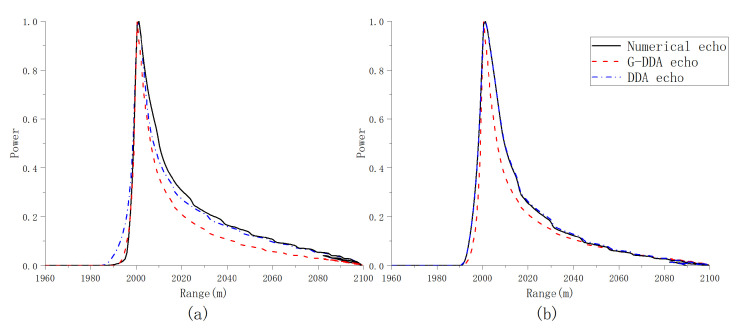
Comparison of multilooked power waveforms in typical airborne scenarios: (**a**) $\left(\mu ,\psi_{ac},\psi_{al}\right)=\left(10,10,10\right)\deg$ and (**b**) $\left(\mu ,\psi_{ac},\psi_{al}\right)=\left(0,10,0\right)\deg$.

In Figure 11, the multilook waveforms computed from the different model implementations are shown when $\left(\mu ,\psi_{ac},\psi_{al}\right)=\left(0^{o},0^{o},0^{o}\right)$ and $\left(\mu ,\psi_{ac},\psi_{al}\right)=\left(18^{o},0^{o},18^{o}\right)$. The waveforms have been normalized with respect to the peak power. From Figure 11 (left side), it can be seen that both the DDA echo and G-DDA echo are close to the numerical echo in the absence of mis-pointing. From Figure 11 (right side), it can be noticed that the shape of the G-DDA echo is far from the expected one when the pitch angle $\psi_{al}$ increases, while this does not occur in case of the DDA echo. In Figure 12, the multilook waveforms are computed when the angles are set as $\left(\mu ,\psi_{ac},\psi_{al}\right)=\left(10^{o},10^{o},10^{o}\right)$ and $\left(\mu ,\psi_{ac},\psi_{al}\right)=\left(0^{o},10^{o},0^{o}\right)$. From Figure 12, it can be noticed that while the shape of the trailing edge in numerical echo has a different decay with respect to the DDA echo and G-DDA echo when the roll increases, the DDA echo is closer to the numerical echo.

### 5.3. Analysis of Re-Tracking Precision

This section evaluates the performance of the proposed re-tracking algorithms for the numerical waveforms affected by speckle noise. To simulate the real echo, speckle noise is added to the multilook waveform (33) following the approach described in [12,14,22]. Consequently, the noisy echo y(t) can be obtained by

(42) $$y\left(t\right)=\sum_{k=1}^{N}P\left(t_{r}-2\delta_{r}\left(f_{d}\right)/c,k\right)q\left(t_{r}-2\delta_{r}\left(f_{d}\right)/c,k\right),$$

where $q\left(t_{r}-2\delta_{r}\left(f_{d}\right)/c,k\right)$ is gamma-distributed, multiplicatively independent, and identically distributed speckle noise expressed as $\Gamma \left(L,1/L\right)$.

The normalized echo used for parameter estimation depends on the unknown parameter vector $\theta_{5}={\left(\mathrm{SWH},\tau ,\mu ,\psi_{ac},\psi_{al}\right)}^{T}$. The effectiveness of the strategy can be evaluated through the simulation of multilook echoes. The root mean square error (RMSE) of the altimetric parameters is adopted to evaluate the algorithm’s performance. All results presented in this section have been averaged using NMC = 500 Monte Carlo runs. The RMSE of the *i*-th altimetric parameter $\theta_{i}$ is defined as

(43) $$\mathrm{RMSE}\left(\theta_{i}\right)=\sqrt{\frac{1}{N}\sum_{j=1}^{N}{\left[\theta_{i}-{\hat{\theta }}_{i}\left(j\right)\right]}^{2}},i=1,\ldots ,J,$$

where $\theta_{i}$ is the true parameter, ${\hat{\theta }}_{i}\left(j\right)$ is the estimated parameter for the *j*-th waveform, and N=500 is the Number of Monte Carlo simulations.

To verify the effectiveness of the proposed model, a generalized delay–Doppler altimetric model is used for comparison. We replace $\psi_{ac}$ and $\psi_{al}$ with zeros and estimate the two remaining parameters ${\left(\mathrm{SWH},\tau \right)}^{T}$ using the Levenberg–Marquardt algorithm. The resulting estimation strategy is denoted as G-PRA (indicating the generalized altimetric model with two unknown parameters used in the parameterized re-tracking algorithm). The estimations provided by G-PRA need to be compared with those obtained with the proposed algorithm, which is denoted as PRA.

**Figure 13:**
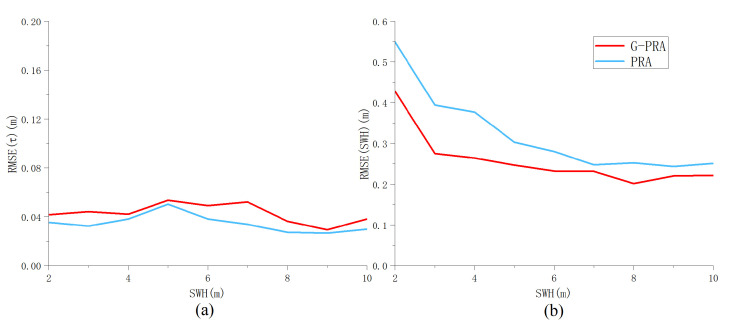
RMSE of (**a**) τ and (**b**) SWH versus SWH in the absence of mis-pointing for the G-PRA and PRA algorithms (Pu = 1, τ = 30 gates, $\psi_{al}=0^{0}$, $\psi_{ac}=0^{0}$).

The first group of experiments corresponds to level flight in the absence of mis-pointing while considering different SWH; the other altimetric parameters are set as ${\left(P_{u},\tau ,\mu ,\psi_{ac},\psi_{al}\right)}^{T}={\left(1,30\;\mathrm{gates},0^{o},0^{o},0^{o}\right)}^{T}$. The parameter RMSEs obtained by the algorithm are shown in Figure 13, and the figure shows similar performance for the G-PRA and PRA algorithms. Thus, the results demonstrate that there is not much performance reduction in estimating τ and SWH in the absence of mis-pointing. Additionally, the $\mathrm{RMSE}\left(\mathrm{SWH}\right)$ is a decreasing function of SWH.

**Figure 14:**
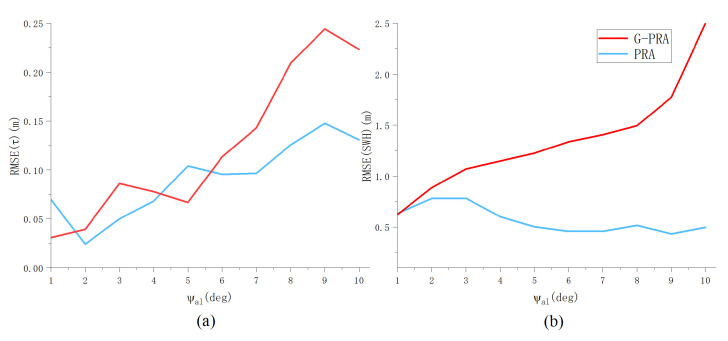
RMSE of (**a**) τ and (**b**) SWH versus $\psi_{al}$ for the G-PRA and PRA algorithms (Pu = 1, τ = 30 gates, SWH = 2 m, $\psi_{ac}=0^{0}$).

**Figure 15:**
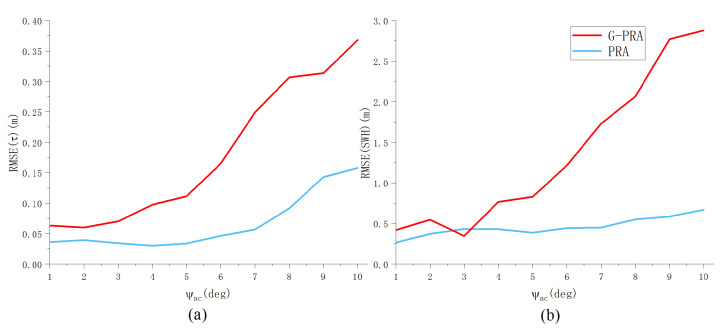
RMSE of (**a**) τ and (**b**) SWH versus $\psi_{ac}$ for G-PRA and PRA algorithms (Pu = 1, τ = 30 gates, SWH = 2 m, $\psi_{al}=0^{0}$).

The second set of experiments aimed to investigate the performance of parameter estimation for oblique flight with varying mis-pointing angles $\psi_{al}$ and $\psi_{ac}$. The remaining altimetric parameters are set as ${\left(\mathrm{SWH},P_{u},\tau ,\mu \right)}^{T}={\left(2\;m,1,30\;\mathrm{gates},6^{o}\right)}^{T}$. Figure 14 demonstrates that the proposed PRA algorithm leads to a smaller $\mathrm{RMSE}\left(\tau \right)$ compared to G-PRA in the presence of $\psi_{ac}$. As $\psi_{ac}$ increases, the difference in $\mathrm{RMSE}\left(\tau \right)$ and $\mathrm{RMSE}\left(\mathrm{SWH}\right)$ obtained by the two algorithms becomes larger. However, the improvement is less in the presence of $\psi_{al}$, as shown in Figure 15. This property can be explained by the fact that the cross-track mis-pointing angle has a greater influence on the waveform shape compared to $\psi_{al}$. In addition, It can be observed that as the mis-pointing angle increases, the RMSE of τ obtained by the PRA algorithm initially remains at a low level and then gradually increases. This may be attributed to the non-negligible error in model approximation. The mis-pointing angle in airborne scenarios is typically not greater than 10 degrees, indicating that the estimation accuracy of this method falls within an acceptable range.

## 6. Conclusions

In order to perform complex navigation tasks, high-precision altimetry on highly mobility platforms is attracting growing attention. However, the waveform re-tracking methods proposed in previous works cannot be applied to highly mobility platforms, which may have a large antenna mis-pointing angle and significant vertical movement. To improve the estimation accuracy of waveform re-tracking, in this paper we propose a novel semi-analytical waveform model and signal processing method for SAR altimeters with vertical movement and large antenna mis-pointing angles. Our main conclusions are as follows:

(1) An analytical expression that considers mis-pointing angles, circular antenna patterns, and vertical velocity is introduced for the echo model. The proposed model shows that the across-track mis-pointing angle affects the shape and the amplitude of the altimetric echo, whereas the along-track mis-pointing angle mainly affects the amplitude of the echo. When we consider $\xi \leq 20^{\circ }$ ($\xi <\theta_{3\mathrm{dB}}/2$), the expansion order m=4 is adequate to achieve the required level of error, which means that the global NQE is less than $10^{-10}$.
(2) A novel delay compensation method based on sinc interpolation is proposed to obtain the multilook echo in order to optimally handle non-integer delays and maintain the signal frequency characteristics. Compared with the generalized model, the proposed semi-analytic model is closer to the fully numerical waveform at different values of mis-pointing angle $\psi_{ac}$ and $\psi_{al}$, especially when the roll angle $\psi_{ac}$ is large.
(3) A five-parameter estimation strategy using the least squares procedure is proposed for SAR altimeters with vertical movement. The performance of the proposed model is evaluated using simulated data for typical airborne scenarios, analyzing the influence of different mis-pointing angles and flight path angle on parameter estimation. When the mis-pointing angles are within 10 degrees, the RMSE of τ obtained by the re-tracking method fitted by the proposed model is less than 0.2 m, and increases more slowly compared to the one fitted by the generalized model.

In February 2024, a more in-depth airborne radar altimeter experiment will be conducted in southern China. The model and re-tracking methods proposed in this paper will be validated against real data collected during this experiment. In addition, future research will focus on exploring the optimal performance of the estimation strategies under various antenna mis-pointing angles; it is worth considering a further modification to the strategy to involve fewer parameters in iterative updates.

## Figures and Tables

**Table 1:** Simulation parameters.

Parameter	Value
Frequency	2.95 GHz
Wavelength (λ)	0.1 cm
Bandwidth (B)	100 MHz
Mean flight altitude (*h*)	2000 m
Pulse repetition frequency (PRF)	5000 Hz
3dB Antenna beam width ($\theta_{3\mathrm{dB}}$)	$40^{\circ }$
Velocity ($v_{s}$)	100 m/s
Pulse per burst	100 pulses

## Data Availability

Data are contained within the article.
